# The role of LOXL2 in tumor progression, immune response and cellular senescence: a comprehensive analysis

**DOI:** 10.1007/s12672-024-01107-9

**Published:** 2024-06-26

**Authors:** Chen Ye, Sihan Jiang, Tanlun Zeng, Shaohui He, Jinjin Cao, Jianru Xiao

**Affiliations:** 1 School of Health Science and Technology, University of Shanghai for Science and Technology, 516 Jungong Road, Shanghai, 200093 China; 2 Spinal Tumor Center, Department of Orthopedic Oncology, Shanghai Changzheng Hospital, Naval Medical University, 415 Fengyang Road, Shanghai, 200003 China; 3 Graduate School, Naval Medical University, 800 Xiangyin Road, Shanghai, 200433 China; 4 Clinical Cancer Institute, Center for Translational Medicine, Naval Medical University, 800 Xiangyin Road, Shanghai, 200433 China

**Keywords:** LOXL2, Pan-cancer analysis, Prognosis, Immunity, Cellular senescence

## Abstract

*LOXL2*, an enzyme belonging to the *LOX* family, facilitates the cross-linking of extracellular matrix (ECM) elements. However, the roles of the *LOXL2* gene in mechanisms of oncogenesis and tumor development have not been clearly defined. In this pan-cancer study, we examined the notable disparity in *LOXL2* expression at the mRNA and protein levels among various cancer types and elucidated its interconnected roles in tumor progression, mutational profile, immune response, and cellular senescence. Apart from investigating the hyperexpression of *LOXL2* being related to poorer prognosis in different types of tumors, this study also unveiled noteworthy connections between *LOXL2* and genetic mutations, infiltration of tumor immune cells, and genes in immune checkpoint pathways. Further analysis revealed the participation of *LOXL2* in multiple pathways related to cancer extracellular matrix remodeling and cellular senescence. Moreover, our investigation uncovered that the knockdown and inhibition of *LOXL2* significantly attenuated the proliferation and migration of PC-9 and HCC-LM3 cells. The knock-down and inhibition of *LOXL2* enhanced cellular senescence in lung and liver cancer cells, as confirmed by SA-β-Gal staining and quantitative RT-PCR analyses. This comprehensive analysis offers valuable insights on the functions of *LOXL2* in different types of cancer and its role in regulating the senescence of cancer cells.

**Supplementary Information:**

The online version contains supplementary material available at 10.1007/s12672-024-01107-9.

## Introduction

Globally, tumors have become a major disease threatening people’s health [1]. Despite substantial progress in the field of cancer treatment, the attainment of curative treatment remains elusive, resulting in persistently high mortality rates [2]. Targeted therapy and immunotherapy have reshaped the therapeutic landscape for cancer patients [3]. However, the therapeutic effects of these treatments exhibit significant variability among individuals and tumor types, thereby highlighting the existing disparity in gene mutation abundance and heterogeneity of the immune microenvironment [4, 5]. Hence, it is imperative to clarify the molecular pathogenesis and immune composition of tumors and conduct analytical validation of universal biomarkers.

The tumor microenvironment (TME) pertains to the internal surroundings that impact the progression and survival of tumor cells [6]. Cells synthesize and release a network of macromolecules, known as the extracellular matrix (ECM). Tumor cells, immune cells, stromal cells, and ECM components make up the TME [7]. The deposition and remodeling of ECM serve as the primary step and biochemical foundation for tumor malignant behaviors and immune cell infiltration [4, 8]. The roles of cellular senescence in tumor cell behaviors are controversial, senescent cells block tumor growth while maintaining a metabolically active state. Despite slowing down tumor progression, cellular senescence encourages metastasis, alters the TME, and affects treatment outcomes [9]. The innate immune system interacts with tumors in TME and plays a dual role in abnormal cellular senescence and elimination. This process involves the participation of CD4 + T cells, macrophages, neutrophils, and natural killer cells [10]. Therefore, it is necessary to find key molecules co-regulating immunity, senescent tumor cell and ECM.

Lysyl oxidase-like-2 (*LOXL2*), a copper-dependent amine oxidase, is a member of the *LOX* family [11]. A major role of *LOXL2* is to facilitate and stabilize the cross-linking process of elastin and collagen within the ECM. Additionally, *LOXL2* has been mainly researched in individual tumors, including breast cancer [12], hepatocellular carcinoma [13], non-small cell lung cancer [14], cervical cancer [15], colon cancer [16], and esophageal cancer [17], and several studies have found its association with tumor progression, metastasis, chemo-radiotherapy resistance, and worse prognosis [18–21]. However, progress towards humanized *LOXL2* targeted drugs is not as expected [22, 23]. During replicative and stress-induced senescence, fibroblasts and epithelial cells exhibited elevated levels of *LOXL2* expression, whereas its occurrence in oncogene-induced cellular senescence was rarely reported [24, 25]. Besides, the pathological effects and function of *LOXL2* are underrecognized, and systematic pan-cancer analysis for *LOXL2* is still lacking.

This study aimed to comprehensively examine the landscape of *LOXL2* in pan-cancer, encompassing its expression level, prognostic value, genetic alteration, and immune infiltration. Additionally, we validated the impacts of *LOXL2* on tumor proliferation, migration, and cellular senescence in vitro. The ultimate objective was to assess the potential of *LOXL2* to be a prognostic and diagnostic biomarker, and afford new thought for personalized therapy.

## Result

### The expression of LOXL2 in *pan cancer*

**Fig. 1:**
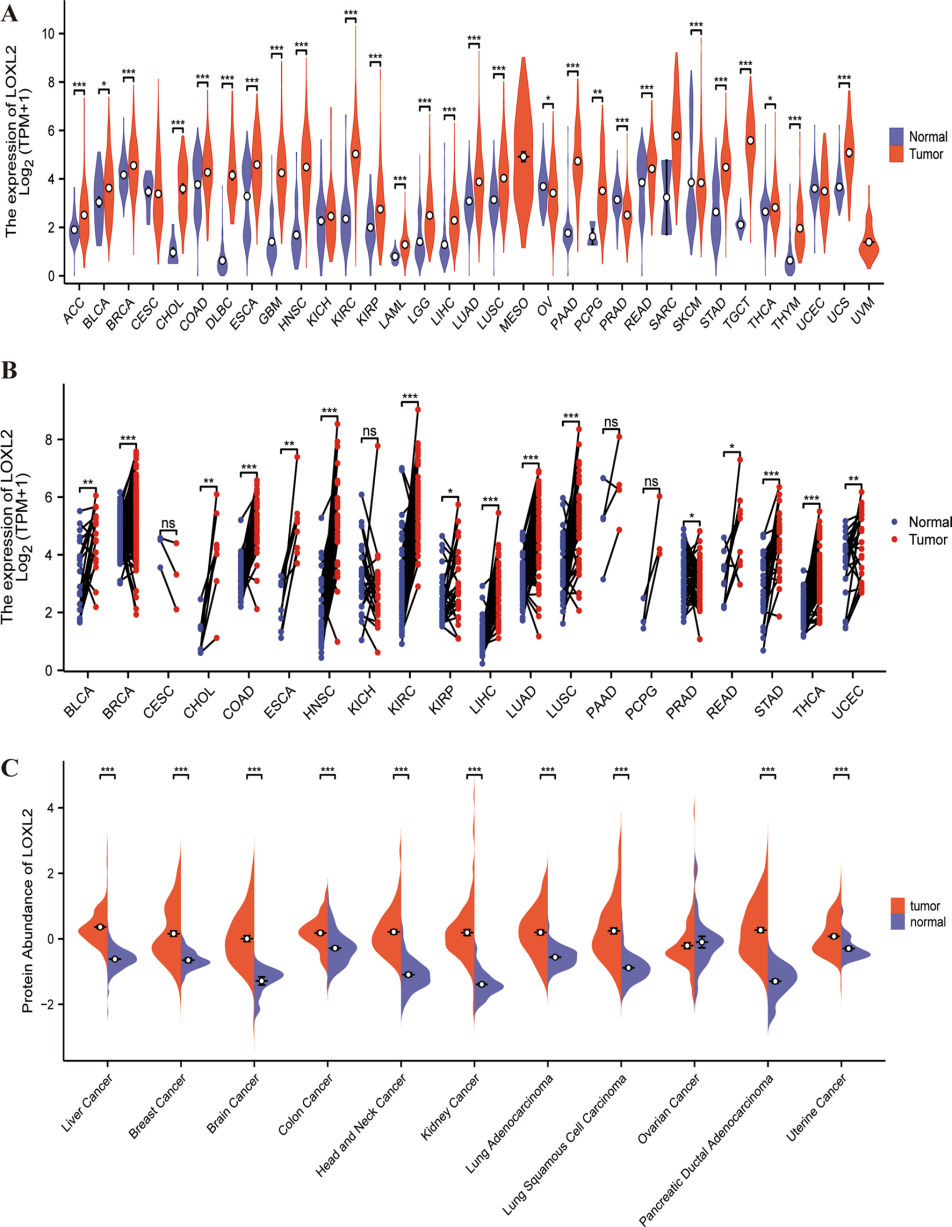
**A** For unpaired normal and tumor samples, mRNA expression levels of *LOXL2* in different cancers were analyzed via the TCGA database, MESO and UVM lack normal tissue data; **B** Protein expression of *LOXL2* in different normal and tumor tissues was analyzed via the CPTAC database; **C** The IHC staining results of *LOXL2* expression in lung, breast, and liver cancers were obtained via the HPA database. * p < 0.05, **p < 0.01, and ***p < 0.001

TCGA data revealed that unpaired tissue mRNA expression of *LOXL2* was significantly higher in tumor tissues than in normal tissues, in ACC, BLCA, BRCA CHOL, COAD, DLBC, ESCA, GBM, HNSC, KIRC, KIRP, LAML, LGG, LIHC, LUAD, LUSC, PAAD, PCPG, READ, SKCM, STAD, TGCT, THCA, THYM, and UCS, besides being down-expressed in OV and PRAD (Fig. 1A). In paired tissue mRNA expression of pan-cancer, significantly increased expression of *LOXL2* was found in tumor tissues of BLCA, BRCA, CHOL, COAD, ESCA, HNSC, KIRC, KIRP, LIHC, LUAD, LUSC, READ, STAD, THCA, and UCEC compared with their normal counterparts (Figure S1A). Additionally, the results of analysis in the CPTAC dataset showed that *LOXL2* protein expression had a noteworthy increase in tumor tissues of liver cancer, breast cancer, brain cancer, colon cancer, head and neck cancer, kidney cancer, lung adenocarcinoma, ovarian cancer, pancreatic ductal adenocarcinoma, and uterine cancer, compared with corresponding normal samples (Fig. 1B). Based on the HPA database, the results of IHC in lung, breast, and liver cancer tissues and corresponding normal tissues revealed that *LOXL2* protein was predominantly localized in the nucleus and ECM (Fig. 1C). Besides, the results of analysis in the TCGA database indicated that *LOXL2* expression was markedly increased in BLCA, GBMLGG, LIHC, LUAD, and UVM with higher clinical or pathological stages (Figure S1B). Overall, a higher level of *LOXL2* expression has been detected in tumor tissues, compared to normal tissues, indicating that *LOXL2* may contribute to tumor pathogenesis through multiple potential mechanisms.

### Prognostic Signature of LOXL2

**Fig. 2:**
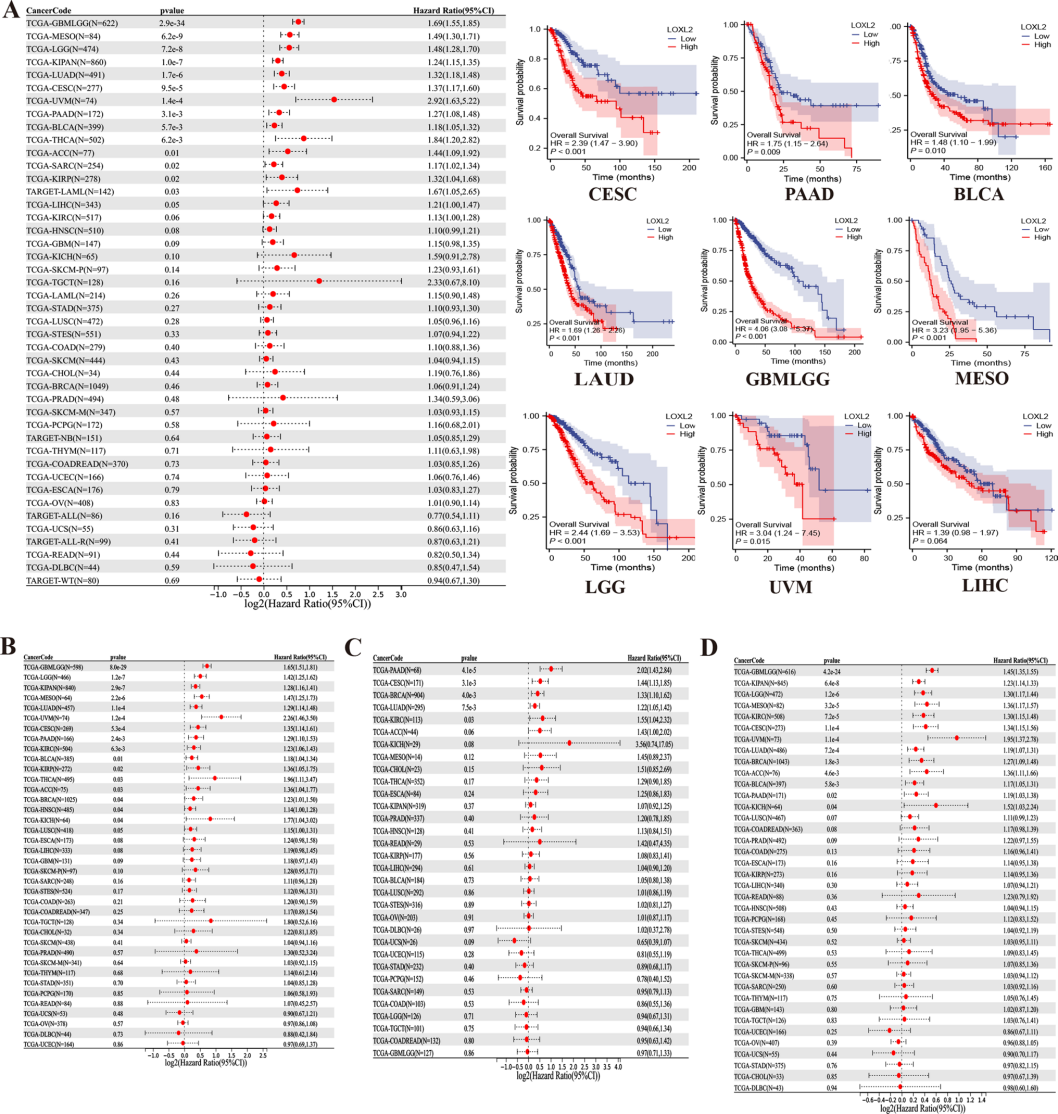
**A** The relationship between *LOXL2* expression and OS was analyzed using the univariate Cox regression method in pan-cancer. In the TCGA database, Kaplan–Meier methodology was used to compare the expression of *LOXL2* in different cancer types, showing OS curves for high and low expression; **B**–**D** The association between *LOXL2* expression and DSS, DFI, and PFI was analyzed using univariate Cox regression analysis

In order to examine the prognostic value of *LOXL2* in pan-cancer patients, we analyzed its expression in relation to patients’ outcomes, concentrating on overall survival (OS), progression-free interval (PFI), disease-free interval (DFI), and disease specific survival (DSS). The results revealed that high-expressed *LOXL2* was related to poor OS in GBMLGG, MESO, LGG, KIPAN, LUAD, CESC, UVM, PAAD, BLCA, THCA, ACC, SARC, KIRP, LAML, and LIHC. In patients with CESC, PAAD, BLCA, LUAD, MESO, LGG, UVM, and LIHC, elevated LOXL2 expression indicated decreased survival time according to Kaplan–Meier survival analysis (Fig. 2A), and non-significant correlations were shown in Figure S2. *LOXL2* expression was correlated with DSS in various cancers, including LUAD, GBMLGG, and other 15 types of cancer (Fig. 2B). It also revealed that *LOXL2* expression was significantly associated with DFI in 13 types of cancers (Fig. 2C). Moreover, the expression of LOXL2 was remarkably related to PFI in 24 types of cancer (Fig. 2D). These results suggested that *LOXL2* might be a useful biomarker in predicting patient prognosis, especially in LUAD, CESC, PAAD, and ACC, and the contribution of *LOXL2* in tumor progression is yet to be fully elucidated.

### Genetic alteration of LOXL2

**Fig. 3:**
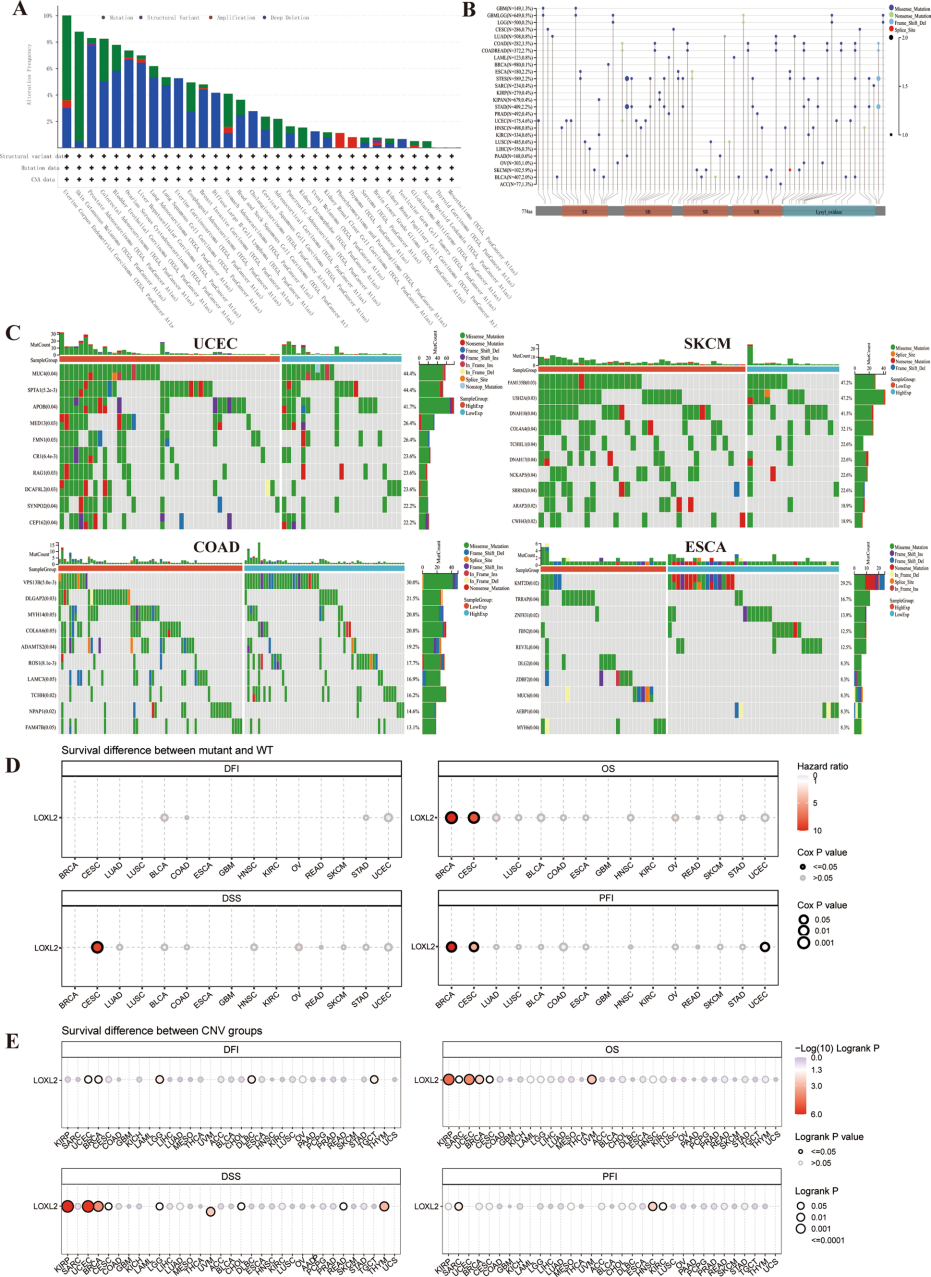
**A** The mutation profile of *LOXL2* revealed “Deep Deletion” was the major type of mutation by TCGA; **B** The mutational landscape of *LOXL2* across various cancers was examined by TCGA; **C** Genetic alteration disparities in UCEC, SKCM, COAD, and ESCA with varying expression levels of *LOXL2* by TCGA; **D**, **E** The correlation of survival with *LOXL2* SNV and CNV in pan-cancer via GSCA.

It is acknowledged that the accumulation of genetic mutations is a key factor of human cancer and also a feature of cellular senescence [9, 26]. The results of analysis in cBioPortal showed that genetic alteration types and frequencies of *LOXL2* differ in multiple cancers. The main genetic alteration type of LOXL2 in pan-cancer is “Deep Deletion” and the top 3 cancer types with high alteration frequencies were UCEC, SKCM, and PRAD. The *LOXL2* gene alterations were all “Deep Deletion” in UCS, DLBC, CHOL, UVM, and TGCT. In PCPG and THYM, the LOXL2 gene alterations were all “Amplification” (Fig. 3A).

LOXL2 mutates extensively in various cancer species (Fig. 3B). The top 3 high-frequency mutations were 5.9% in SKCM, 4.6% in UCEC, and 3.5% in COAD (Fig. 3B). Furthermore, we evaluated the differences in gene alteration frequencies of tumors with higher expression of *LOXL2*, among the forementioned four types of tumors (UCEC, SKCM, COAD, and ESCA), *APOB*, *USH2A*, *VPS13B,* and *KMT2D* had the highest alteration frequency in the above cancer types (Fig. 3C). Besides, cancer patients with *LOXL2* alternations of stable nuclear variant (SNV) had worse OS and PFS than those with no alterations in BRCA and CESC, while the copy number variation (CNV) of *LOXL2* is associated with a worse prognosis in KIRP, UCEC, and BRCA (Fig. 3D, E). The above results show that the genetic alteration of *LOXL2* may play a crucial role in tumorigenesis and tumor progression.

### Immunological role of LOXL2

**Fig. 4:**
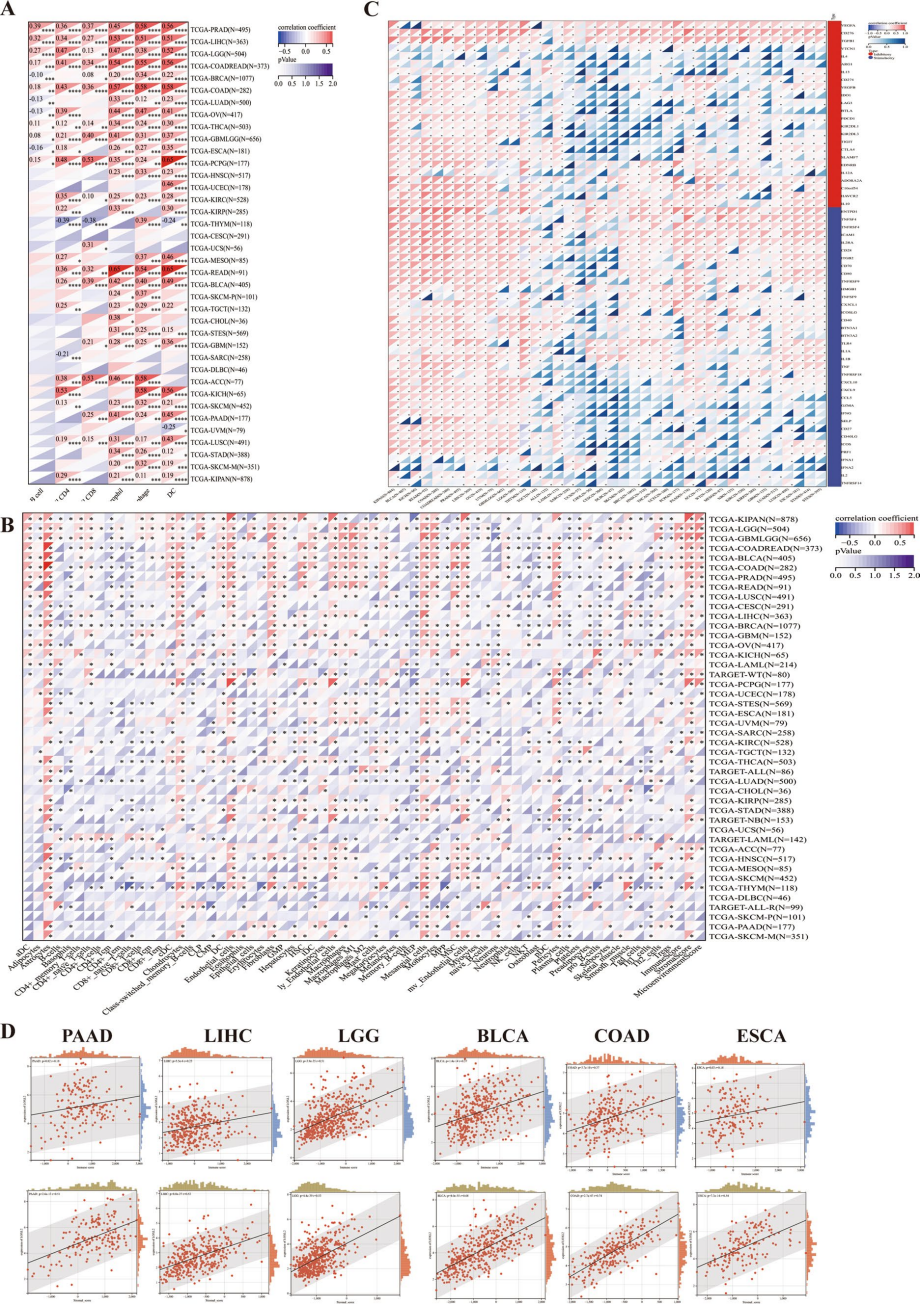
**A** Heatmap showing correlations of *LOXL2* expression with immune infiltration level in various TCGA cancer types using TIMER; **B** Heatmap of relationships of *LOXL2* expression with infiltrated cells in various TCGA cancer types using xCell; **C** The connection between *LOXL2* expression and genes in immune checkpoint pathways; **D** The correlation of *LOXL2* expression and stromal scores, immune scores in pan-cancer via ESTIMATE and TCGA databases

The relationship between immune cell infiltration and *LOXL2* expression was examined using the xCell and TIMER algorithms. The correlation between immune cell infiltration, specifically B cell in 12 types, CD4 + T cell in 23 types, CD8 + T cell in 18 types, neutrophil in 29 types, macrophage in 30 types, and dendritic cell in 31 types, and *LOXL2* expression was examined in 38 types of tumors using the TIMER method (Fig. 4A). Our analysis revealed associations between the majority of the 64 immune cell subtypes and *LOXL2* expression in various tumor types (Fig. 4B). The role of immune checkpoint genes in tumor immunotherapy is crucial [27]. We conducted investigations to examine the association between the expression of *LOXL2* and the genes in immune checkpoint pathways [28]. Our findings indicated a significant relationship between *LOXL2* expression and the majority of the genes in immune checkpoint pathways, like *VEGFA*, *CD276*, *TGFB1,* and *IL10* in pan-cancer. Furthermore, we observed a positive correlation between *LOXL2* expression and genes in major checkpoint pathways in KIPAN, BLCA, KICH, READ, COAD, PRAD, LIHC, OV, UVM, GBMLGG, and LGG (Fig. 4C). Consequently, the immune infiltration scores, stromal scores and Pearson correlation of *LOXL2* expression in pan-cancer have been calculated via the ESTIMATE algorithm. Among them, the *LOXL2* pattern has a positive correlation with immune scores, and stromal scores in PAAD, LIHC, LGG, BLCA, COAD, and ESCA (Fig. 4D). *LOXL2* expression was positively associated with the number of CD4 + T and CD8 + T cells. The stromal scores in 31 tumors also have a positive correlation with the RNA levels of *LOXL2* via the xCell algorithm (Fig. 4B). TME associated fibroblast, pericyte, and endothelial cell also showed significant negative associations in multiple cancers (Fig. 4B). Above all, *LOXL2* has the potential to regulate stromal cells, modify the tumor immune microenvironment, and impact the response of immunotherapy in multiple tumors.

### Enrichment analysis of LOXL2-related genes

**Fig. 5:**
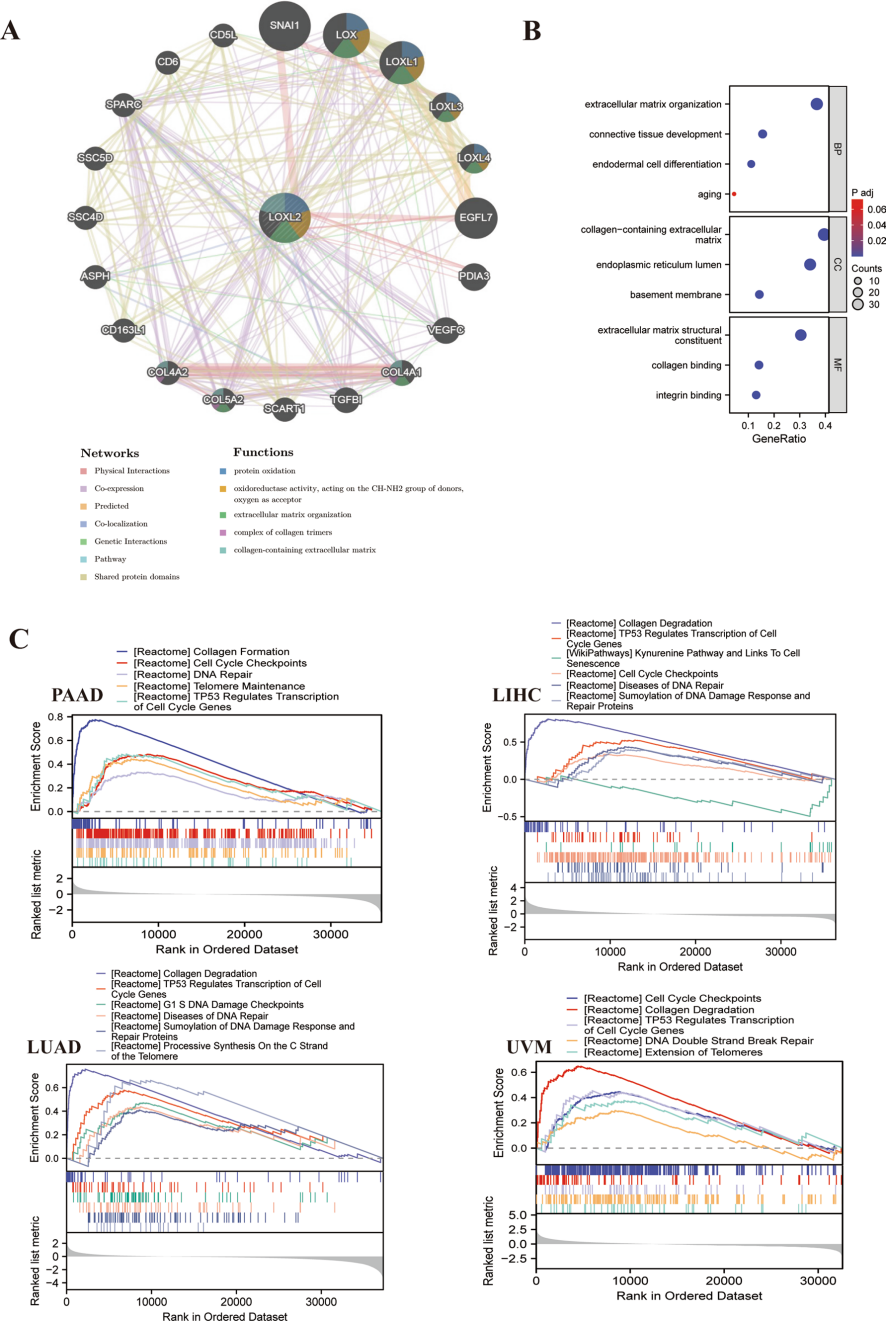
**A** Through GeneMANIA, *LOXL2* and its molecular partners form a PPI network; **B** KEGG enrichment analysis of the gene set from GEPIA2; **C** GSEA analysis of *LOXL2* in different cancers. Various colored curves represent distinct functions or pathways controlled by various types of cancer. Positive regulation is indicated by peaks on the ascending curve, while negative regulation is indicated by peaks on the descending curve.

In order to comprehensively elucidate the protein co-expression, potential biological functions, and signaling pathways associated with *LOXL2*, an analysis was performed using GeneMANIA and GSEA. The protein–protein interaction (PPI) network generated from GeneMANIA uncovered interactions between *LOXL2* and *SNAI1, LOX, LOXL1, LOXL3, EGFL7,* and *VEGFC* (Fig. 5A). Among them, there was ample evidence that *SNAI1* has a physical interaction with *LOXL2*. Meanwhile, the functional analysis indicated that *LOXL2* and its molecular partners were primarily focused on protein oxidation, oxidoreductase function, acting on the CH-NH2 group of donors, oxygen as acceptor, extracellular matrix organization, complex of collagen trimers, and collagen-containing extracellular matrix (Fig. 5A).

The top 100 genes with the most comparable expression patterns to *LOXL2* in pan-cancer were extracted from GEPIA2. The above 100 genes were closely related to cell migration, adhesion, and cellular senescence based on the KEGG enrichment analysis (Fig. 5B). Based on the median *LOXL2* mRNA expression level, TCGA samples were divided into high and low expression levels for *LOXL2*, and enriched gene collections were identified. As a result of the GSEA analysis, the high *LOXL2* expression group showed altered cancer-associated (Cell Cycle, *TP53* Regulation), TME-associated (Collagen Degradation) and cellular senescence-associated (DNA Repair, Telomere Maintenance, Metabolic pathway) pathways in PAAD, LIHC, LUAD, and UVM (Fig. 5C).

### LOXL2 expression effects cell proliferation and migration

**Fig. 6:**
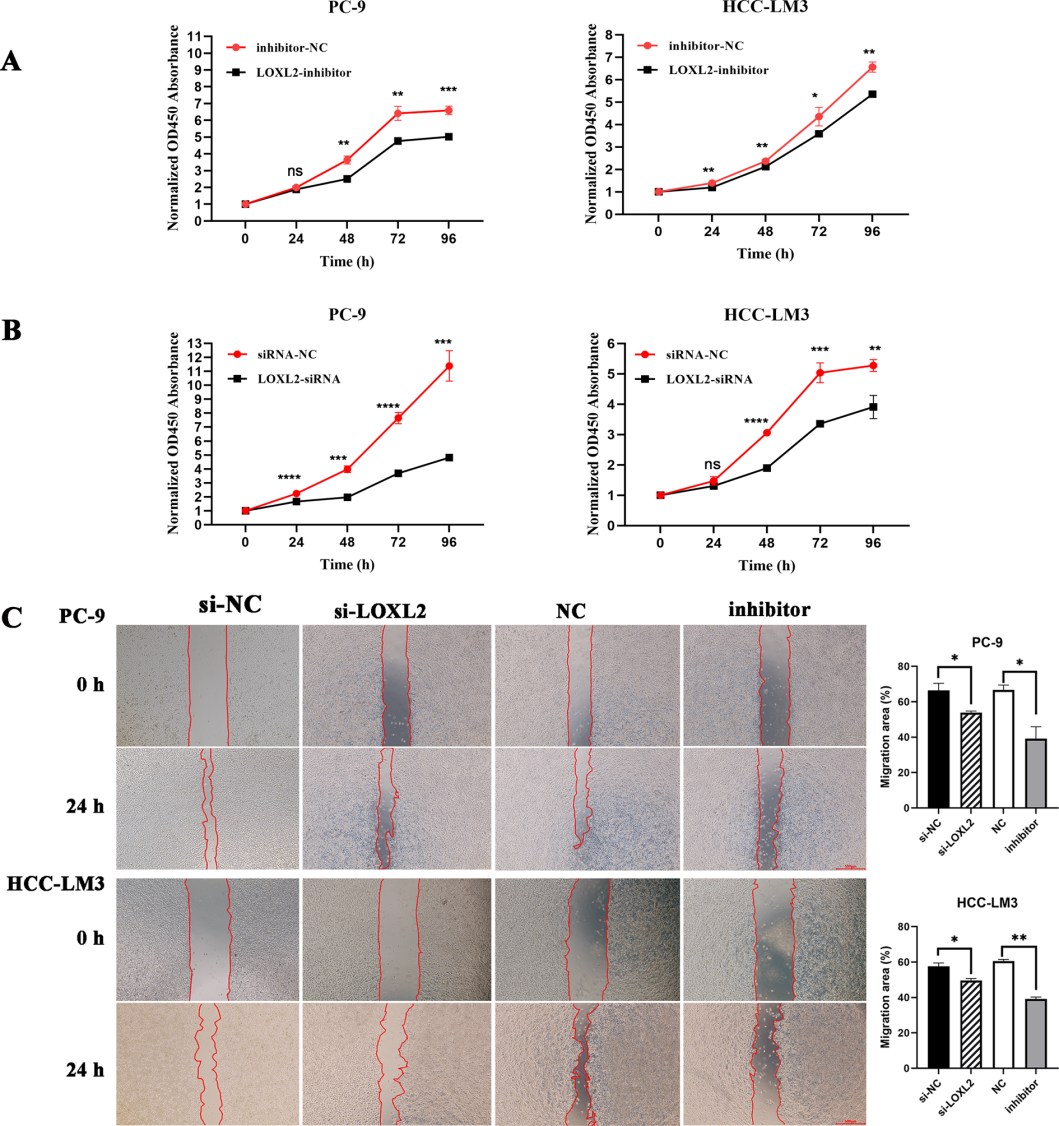
**A** The impact of *LOXL2* on cell proliferation in PC-9 and HCC-LM3 cells was assessed using the CCK8 assay at 24, 48, 72, and 96 h; **B** The migration ability of PC-9 and HCC-LM3 cells was compromised by both *LOXL2* knockdown and the *LOXL2* inhibitor. Scale bar = 500 µm. The statistical findings from the scratch wound-healing assays at 24 h are displayed as the average plus standard deviation (three replicates). * P < 0.05; ** P < 0.01; *** P < 0.001; **** P < 0.0001

According to the previous bioinformatic data, the occurrence and development of tumors in pan-cancer are strongly correlated with the expression of *LOXL2*. The effects of *LOXL2* on proliferation and migration were examined in PC-9 and HCC-LM3 cells using CCK-8 and wound healing assays. The expression of *LOXL2* was down-regulated by siRNA and *LOXL2* inhibitors in PC-9 and HCC-LM3 cells (Figure S3). PC-9 and HCC-LM3 cells were significantly impeded in proliferation by decreasing *LOXL2* expression in the CCK-8 assay at 48 h, 72 h and 96 h. This finding was further supported by the administration of a *LOXL2* inhibitor (1 µM) (Fig. 6A). Furthermore, the wound healing experiments demonstrated that *LOXL2* knockdown impaired the migration of PC-9 and HCC-LM3 cells. Consistent outcomes were observed in wound healing assays following treatment with the *LOXL2* inhibitor (1 µM) for 48 h (Fig. 6B). The findings suggested that *LOXL2* has the ability to suppress the growth and migration of tumor cells.

### Down-regulation of LOXL2 promotes cellular senescence

*LOXL2* has been associated with replicative and stress-induced senescence in previous studies of skeletal muscle and lung diseases, whereas it is rarely reported in oncogene induced cellular senescence [24, 25]. Moreover, GSEA results showed that *LOXL2* was involved in “DNA Repair”, “Telomere Maintenance” and “Collagen Degradation”, which were closely related to cellular senescence.

**Fig. 7:**
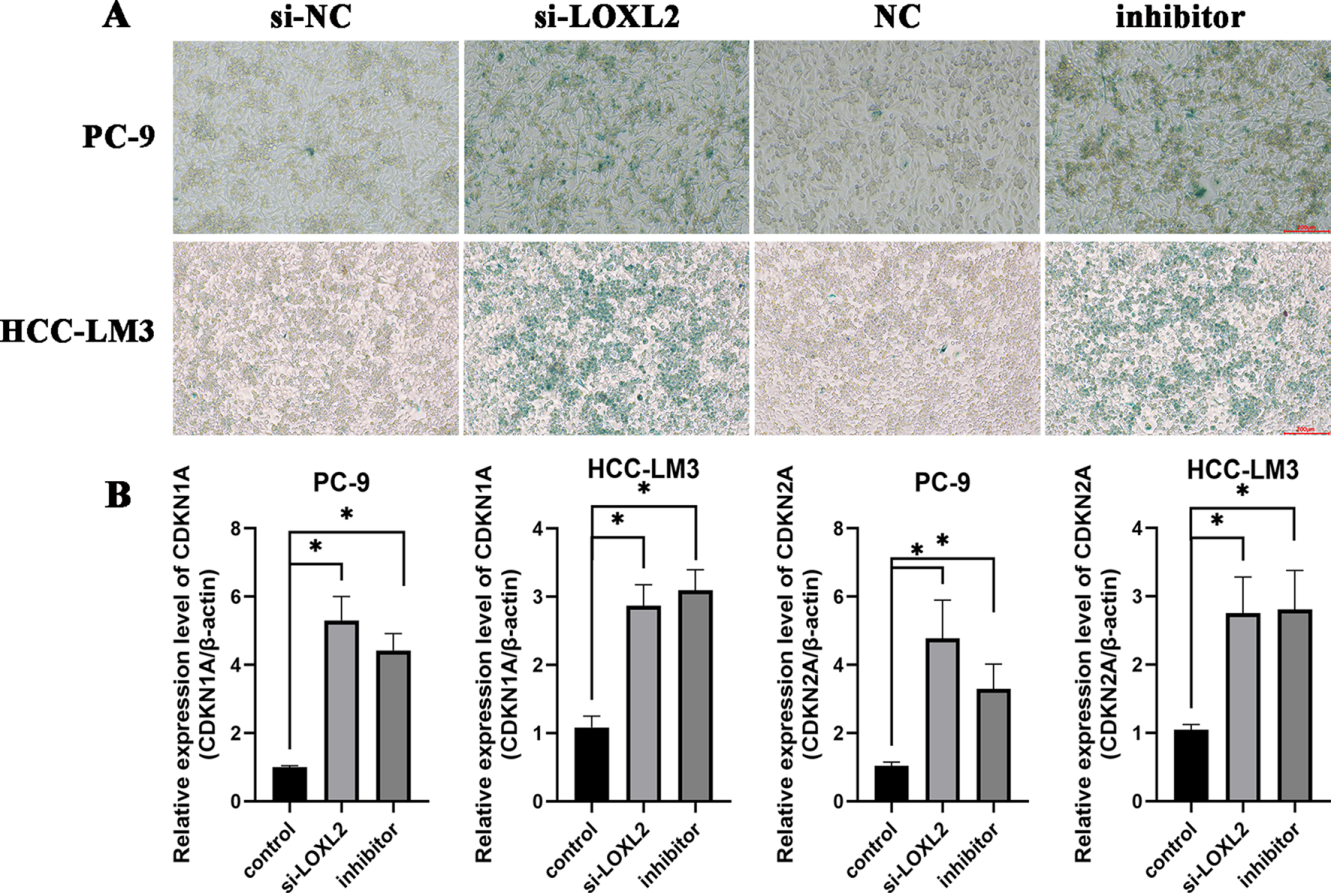
**A** Cellular senescence was detected by SA-β-gal staining. Scale bar = 200 µm; **B** Cellular senescence-related gene expression in PC-9 and HCC-LM3 cells transfected with siRNA-LOXL2 and inhibitor, and control siRNA. * P < 0.05

To examine the influence of *LOXL2* on cellular senescence in tumors, we performed β-galactosidase (SA-β-gal) staining and real-time quantitative PCR assays in lung cancer and liver cancer cells. The SA-β-Gal staining results indicated that knock-down of *LOXL2* facilitated cellular senescence in lung and liver cancer cells (Fig. 7A). Furthermore, knockdown of *LOXL2* resulted in elevated levels of the cellular senescence markers *CDKN1A* and *CDKN2A* (positively correlated with cellular senescence) (Fig. 7B). These findings were consistently observed after treatment with *LOXL2* inhibitor. Therefore, our results suggest that L*OXL2* may depress cellular senescence in multiple tumors.

## Method

### Pan-cancer expression

To compare the *LOXL2* (ENSG00000134013) expression in tumor and normal tissues, we extracted the mRNA expression data of pan-cancer from the Cancer Genome Atlas (TCGA) database via the GDC portal (https://portal.gdc.cancer.gov/repository). The data were converted into relative expression (transcripts per million /TPM) and the expression value was transformed into log2(data + 1) form. The R software package “ggplot2” (version 3.3.3, https://cran.r-project.org/web/packages/ggplot2/index.html) was utilized to display *LOXL2* expression levels in cancer and adjacent normal tissues. The significance of the difference in expression between tumor and normal tissues was assessed through the Wilcoxon rank sum test. Additionally, the protein expression of *LOXL2* in normal and primary tumor tissues was examined through the Clinical Proteomic Tumor Analysis Consortium (CPTAC) module within the UALCAN portal [29] (https://ualcan.path.uab.edu/analysis-prot.html). Besides, we present an overview of *LOXL2* protein expression across lung, breast, and liver cancers from the immunohistochemistry (IHC) pictures in the HPA database (https://www.proteinatlas.org/).

### Prognostic signature

The survival data of LOXL2 in 44 tumors was obtained from the UCSC [30] (https://xenabrowser.net/) of TCGA PANCAN with TARGET PANCAN and GTEx (N = 19,131, G = 60,499). We also excluded the cancers with less than 10 samples in a single cancer, and finally obtained the expression data and the overall survival data of all samples in the 44 cancers, as shown above. The univariate Cox proportional hazards regression model was utilized to examine the association between gene expression and clinical outcome in the R software package “survival” (version 3.2–7). The prognostic significance of *LOXL2* expression was assessed using a log-rank test. R packages “survminer” and “ggplot2” [31] generated Kaplan–Meier curves and forest plots to present the outcomes of OS, PFI, DFI, and DSS.

### Genetic alteration

To uncover the genetic alteration of *LOXL2*, we utilized the cBioPortal (http://www.cbioportal.org/) database, an open-access database designed for interactive exploration of pan-cancer genomics datasets. The cohort of “TCGA Pan-Cancer Atlas Studies” was chosen for analysis. Additionally, we obtained comprehensive cancer genomic data from the UCSC Xena database (https://xena.ucsc.edu/), specifically the TCGA TARGET GTEx Pan-Cancer data, and conducted an analysis on the genomic alteration of *LOXL2* across various cancer types. Moreover, we employed the GDC portal, MuTect2 software [32], and the R software package “maftools” [33] to integrate, calculate, and display the simple nucleotide mutations of UCEC, SKCM, COAD, and ESCA genomic data, which were sourced from the TCGA database. The results of genomic alteration were depicted using bar plots, and the genomic alteration rate was visualized by a lollipop plot. The results of genomic alteration (stable nuclear variant (SNV) and copy number variation (CNV)) correlation with survival were calculated via GSCA [34] (https://guolab.wchscu.cn/GSCA/#/mutation).

### Immune infiltration landscape

The Tumor Immune Estimation Resource (TIMER) [35] is a useful tool for systematically analyzing immune infiltrates within various cancer types. In this study, we employed the TIMER and xCell methods from the R package “IOBR” [36] to examine the connection between *LOXL2* expression and the quantity of immune cells in each patient. Furthermore, we performed a co-expression analysis of *LOXL2* with genes in immune checkpoint pathways (inhibitory (24) and stimulatory (36)) [28] using Spearman correlation, with statistical significance defined as p < 0.05. To further evaluate the relation of *LOXL2* with immune and stromal scores in pan-cancer, we employed R package “ESTIMATE” (version 1.0.13) and the xCell method. To visualize the results, we employed the R packages “limma”, “reshape2” and “RColorBreyer”.

### Gene ontology enrichment analysis

To investigate the intrinsic mechanisms and signaling routes linked to *LOXL2*, we utilized GeneMANIA (http://genemania.org/), an online database [37] renowned for building protein–protein interaction (PPI) networks, to forecast the protein that interacted with *LOXL2* based on fundamental parameters such as co-expression, co-location, and physical interaction. The top 20 genes were identified and employed to construct the PPI network. To uncover the potential molecular partners in pan-cancer, the TCGA dataset was analyzed using the Similar Gene Detection module of GEPIA2 (https://gepia2.cancer-pku.cn/) to identify genes exhibiting expression patterns that closely resemble *LOXL2*. The GO and KEGG databases were utilized for gene and pathway annotation, while the R packages “clusterProfiler” [38] and “ggplot2” were employed to visualize the enrichment results. In addition, we conducted the gene set enrichment analysis (GSEA, https://www.gsea-msigdb.org/gsea/downloads.jsp). High- and low-expression groups of *LOXL2* were defined based on patient mRNA levels, and subsequently subjected to GSEA enrichment analysis using the Rectome database (https://reactome.org/) and Wiki pathways gene sets (http://wikipathways.org/). Pathways that showed significant enrichment were identified using the following criteria: |NES|> 1, p < 0.05, and FDR q < 0.25.

### Cell culture

Human lung cancer cell line PC-9 and human hepatocellular carcinoma cell line HCC-LM3 were acquired from the Cell Bank of China Academy of Sciences (Shanghai, China), and cultured in RPMI1640 and DMEM media, respectively. Transfection of siRNAs was performed using the Lipofectamine™ 3000 kit (Invitrogen, USA), according to the manufacturers’ instructions. All siRNA sequences were designed and synthesized via Sangon Biotech (Shanghai, China). The siRNA sequence targeting *LOXL2* included sense: 5’-GAAACCCTCCAGTCTATTATA-3’; antisense: 5’-TATAATAGACTGGAGGGTCTT-3’. (2-Chloropyridin-4-yl) methanamine hydrochloride (MCE, State of New Jersey, USA) is a selective inhibitor for LOXL2 [39, 40].

### Real-time quantitative PCR

Total RNA was extracted with the RNA fast2000 kit (Fastagen, shanghai, China), reversed-transcribed into complementary DNA (cDNA) using the Reverse Transcription kit (Takara, Dalian, China) following the manufacturer’s protocol. Real-time quantitative PCR was conducted with the Universal SYBR qPCR Master Mix (Vazyme, Nanjing, China) and Bio-Rad CFX96TM Real-time System. The β-actin gene was used as an endogenous control. The experiment was done with three technical replicates. Primer sequences are listed in Table 1.

**Table 1:** The primer sequences utilized in this investigation

Gene name	Forward (F)	Reverse (R)
CDKN2A	GGGTTTTCGTGGTTCACATCC	CTAGACGCTGGCTCCTCAGTA
CDKN1A	TGTCCGTCAGAACCCATGC	AAAGTCGAAGTTCCATCGCTC
β-actin	CATGTACGTTGCTATCCAGGC	CTCCTTAATGTCACGCACGAT

### Cell counting kit-8 (CCK8) assay

Cell proliferation was measured using a CCK-8 kit (NCM Biotech, Suzhou, China). Transfected cells or 1 μM inhibitor treated cells were plated in 96-well plates at a density of 2000–3000 cells/well, and the optical density of the samples was determined at 450 nm with a microplate reader at 0, 24 h, 48 h, 72 h, and 96 h after staining with CCK-8 reagent. Statistical significance was determined using two-tailed Student's t-tests.

### SA-β-galactosidase (SA-β-Gal) staining

The plates were filled with cells at a concentration of 7 × 10^4^ per well in 48-well plates. The experimental groups consisted of control siRNA, siRNA-*LOXL2*, control, and inhibitor (1 μM, seeded at 48 h) Subsequently, the cells underwent SA-β-Gal staining (Beyotime, Beijing, China) following the guidelines provided by the manufacturer. Senescent cells were characterized by blue staining.

### Wound healing assay

Cells transfected or treated with 1 μM inhibitor were quantified and seeded in 6-well plates. Straight lines were drawn on the plate surface using a 200 μL pipette tip. Following two rounds of PBS cleaning, serum-free medium was introduced for subsequent cell culture. The experimental groups consisted of control siRNA, siRNA-*LOXL2*, control, and inhibitor (1 µM). Subsequently, photographs were captured after rinsing the wells with PBS at the 24-h mark, and the migration areas were subsequently analyzed using ImageJ software.

## Discussion

Cancer is the major burden and fatal disease alongside population growth and aging. With the development of new innovative agents and treatment modalities in oncology, clinical practice has greatly benefited over the last few years [41]. The utilization of genetic testing and targeted therapy in a clinical setting demonstrates that the molecular characterization of oncogenes serves as the initial step in the ongoing investigation of underlying mechanisms and intervention strategies [42, 43]. A comprehensive analysis across different types of cancer may uncover shared characteristics and provide therapeutic strategies for cancers with similar features, and pan-cancer analysis has the potential to unveil a universal gene signature across diverse cancer types.

*LOXL2* has been identified as a carcinoma promotor since 2003 [44], for its underlying roles in maturation and remodeling of ECM as an extracellular enzyme. Subsequently, a multitude of studies have elucidated the association between elevated expression of *LOXL2* and diminished overall survival rates, as well as exacerbated clinicopathological attributes of tumors [12, 45, 46]. Furthermore, new roles of *LOXL2* have been identified in the cytoplasm, perinuclear region, and nucleus, most of which are linked to its amine oxidase activity [18, 47–49]. In the extracellular milieu, the presence of *LOXL2* was found to enhance stromal stiffness and facilitate the promotion of adjacent fibroblasts, thereby establishing a supportive niche within the cancer microenvironment [50–52]. This phenomenon is also viable in the case of distant metastasis, as the secretion of *LOXL2* upon reaching distant organs can stimulate the restructuring of the ECM and initiate the development of premetastatic niches [53–55]. However, the efficacy of *LOXL2*-targeted therapy was found to be suboptimal, as evidenced by the unsatisfactory patient outcomes observed in multiple randomized phase II clinical trials involving the humanized *LOXL2* antibody Simtuzumab [22, 23], and more targeted agents and combinational regimens are needed to enter clinical studies [20]. The current understanding of molecular biology in *LOXL2* is not yet complete. Notwithstanding the significance of regulation in the context of cancer, it appears that this is the inaugural pan-cancer analysis of *LOXL2* conducted thus far. By combining data from various types of cancer, we extensively investigated the panorama of *LOXL2* in pan-cancer and elucidated its interconnected role in tumor progression, mutational profile, immune response, and cellular senescence.

Our initial comprehensive examination of *LOXL2* across various cancer types revealed its prevalent overexpression and association with unfavorable clinical results. Depending on this research, *LOXL2* was markedly increased in numerous tumors when analyzing paired (16 types of tumors) and unpaired (27 types of tumors) samples at the mRNA level and 10 tumor types of unpaired protein samples, which aligns with previous reports [20, 21], especially for liver, lung, breast, kidney, uterine, head and neck cancers. Cox and Kaplan–Meier curve analysis indicated a significant association between elevated *LOXL2* expression and an increased incidence of poor OS (15 tumor types), DSS (17 tumor types), DPI (13 tumor types), and PFI (24 tumor types). The findings across multiple types of cancer are in line with previous results obtained from studies focused on a singular type of cancer, and indicating LOXL2 as a possible predictive biomarker for multiple cancers [12–16, 49]. Besides, the secreted characteristics of the *LOXL2* protein determine its promising role in cancer screening and prognostic evaluation, indicating that *LOXL2* can be considered a predictive and prognostic indicator for specific types of malignancies. More samples and high-quality clinical experiments are needed to be conducted.

The occurrence and advancement of cancer result from a sequence of genetic alteration and changes in oncogene molecular feature that are of great significance to guide therapy and monitor therapeutic effects [26]. We examined the genetic alteration of *LOXL2* in various types of cancer. The main genetic alteration type of *LOXL2* in pan-cancer is “Deep Deletion” and UCEC, SKCM, and COAD had the highest mutated frequencies of 5.9%, 4.6%, and 3.5%, respectively. *LOXL2* gene mutations were all “Deep Deletion” in USC, DLBC, CHOL, and UM, but were all “Amplification” in PCPG and THYM. However, a report revealed that *LOXL2* overexpression is more common than mutation [20], follow-up studies are required for specific cancer types. Variations in *LOXL2* expression led to passenger mutations in *APOB, USH2A, VPS13B,* and *KMT2D* genes. Mutations of *APOB, USH2A,* and *KMT2D* were reported to be associated with poor prognosis in several cancer types, and could be considered targets for combination therapy [56–58]. Patients with *LOXL2* alternations of SNV had a worse prognosis than those with no alterations in BRCA and CESC, while the CNV is associated with a worse prognosis in KIRP, UCEC, and BRCA. The presence of *LOXL2* mutations has a substantial effect on the progression of tumors, resulting in tumor heterogeneity. Identifying specific genetic mutations and molecular profiles prior to targeted therapy will maximize cancer patients' benefits [59].

It is less known how *LOXL2* affects the tumor immune milieu. We conducted research to explore the correlations between the expression of *LOXL2* and the immune environment in various types of cancer. The correlation between immune cell infiltration and *LOXL2* expression was examined in 38 types of tumors using the TIMER method. The xCELL method determined the association between the majority of the 64 immune cell subtypes and LOXL2 expression in different tumor types. *LOXL2* expression was positively associated with the number of CD4 + T and CD8 + T cells, indicating that T cell infiltration could be promoted by LOXL2. Different single tumor studies showed disparate effects of *LOXL2* on immune cell infiltration [60, 61]. A reciprocal influence exists between *LOXL2* and immune cells, a more in-depth study may be required to clarify a specific mechanism. *LOXL2* expression was positively correlated with genes in major checkpoint pathways, like *VEGFC, CD276, TGFB1, and IL10*, which may result in an immunosuppression microenvironment. Notably, *LOXL2* expression exhibited a strong correlation with fibroblast, pericyte, and endothelial cells in various malignancies. *LOXL2* protein as an ECM modifying factor, may alter TME by affecting stromal cells, because of its regulatory roles in vasculogenic mimicry and cell adhesion. Therefore, immunotherapy in cancer patients with *LOXL2* overexpression requires more factors for consideration in personalized medicine.

GeneMANIA examined the impact of *LOXL2* on the downstream signaling pathways. Multiple cellular senescence pathways (including DNA Repair, Telomere Maintenance, and Metabolic pathway) were significantly enriched. In the above bioinformatic research, *LOXL2* was proven to have a significant association with the advancement of tumors and cellular senescence, subsequently, we performed CCK-8, wound healing, and SA-β-gal staining assays. Down-regulation of *LOXL2* expression promoted cellular senescence and limited the proliferation and migration abilities of PC9 and HCC-LM3 cells. *LOXL2* was identified as being associated with tumor progression and cellular senescence. *LOXL2* has been reported in the process of senescent morphogenesis in fibroblast and epithelial cells, leading to skeletal muscle fibrosis and lung diseases [24, 25], but is rarely analyzed in tumor research. *LOXL2*-induced cellular senescence was reported for the first time in pan-cancer within the present study, which may aid in related research and the development of new cancer therapies.

## Limitation

Some limitations warrant further deliberation. Our study data is based on public databases, which may be constrained due to inadequate sample size for specific studies. By far, we cannot definitively state that the strong connections we observed in pan-cancer are equally prevalent in every type of cancer. The effects of *LOXL2* on tumor infiltrated immune cells and oncogenic senescence have not yet been elucidated, it eagerly anticipates future exploration of the mechanisms underlying tumor advancement and the transition to a senescent state. The development of therapeutically viable drugs targeting *LOXL2* poses challenges due to the presence of various active mechanisms and barriers associated with inhibiting specific domains. Additional research is necessary to illustrate the underlying mechanism of senescence and further elicit tumorigenesis, thereby optimizing corresponding therapeutic interventions.

## Conclusion

Our initial comprehensive analysis of *LOXL2* across diverse cancer types revealed its prevalent overexpression and association with unfavorable clinical results, subsequent genetic alterations, and immunological context. Furthermore, the roles of *LOXL2* in tumor progression and cellular senescence were partly verified in vitro. Overall, *LOXL2* could be a potential prognostic and diagnostic biomarker, and afford new thought for tumor personalized therapy.

### Supplementary Information

Additional file1 (DOCX 826 KB)

## Data Availability

The original contributions presented in the study are included in the article/Supplementary Material. Further inquiries can be directed to the corresponding author.

## Abbreviations

ACC: Adrenocortical carcinoma
BLCA: Bladder urothelial carcinoma
BRCA: Breast invasive carcinoma
CESC: Cervical squamous cell carcinoma and endocervical adenocarcinoma
CHOL: Cholangiocarcinoma
COAD: Colon adenocarcinoma
DLBC: Lymphoid neoplasm diffuse large B-cell lymphoma
ESCA: Esophageal carcinoma
GBM: Glioblastoma multiforme
HNSC: Head and neck squamous cell carcinoma
KICH: Kidney Chromophobe
KIPAN: Pan-kidney cohort (KICH + KIRC + KIRP)
KIRC: Kidney renal clear cell carcinoma
KIRP: Kidney renal papillary cell carcinoma
LAML: Acute myeloid leukemia
LGG: Brain lower grade glioma
LIHC: Liver hepatocellular carcinoma
LUAD: Lung adenocarcinoma
LUSC: Lung squamous cell carcinoma
MESO: Mesothelioma
OV: Ovarian serous cystadenocarcinoma
PAAD: Pancreatic adenocarcinoma
PCPG: Pheochromocytoma and paraganglioma
PRAD: Prostate adenocarcinoma
READ: Rectum adenocarcinoma
SARC: Sarcoma
SKCM: Skin cutaneous melanoma
STAD: Stomach adenocarcinoma
TGCT: Testicular germ cell tumor
THCA: Thyroid carcinoma
THYM: Thymoma
UCEC: Uterine corpus endometrial carcinoma
UCS: Uterine carcinosarcoma
UVM: Uveal melanoma
