# Supplementary material for: The role of LOXL2 in tumor progression, immune response and cellular senescence: a comprehensive analysis
Source: Discov Oncol. 2024 Jun 26;15:245. doi: 10.1007/s12672-024-01107-9 (PMC11208360; doi:10.1007/s12672-024-01107-9)
Supplement: Supplementary file 1 — Additional file1 (DOCX 826 KB) [file 12672_2024_1107_MOESM1_ESM.docx]

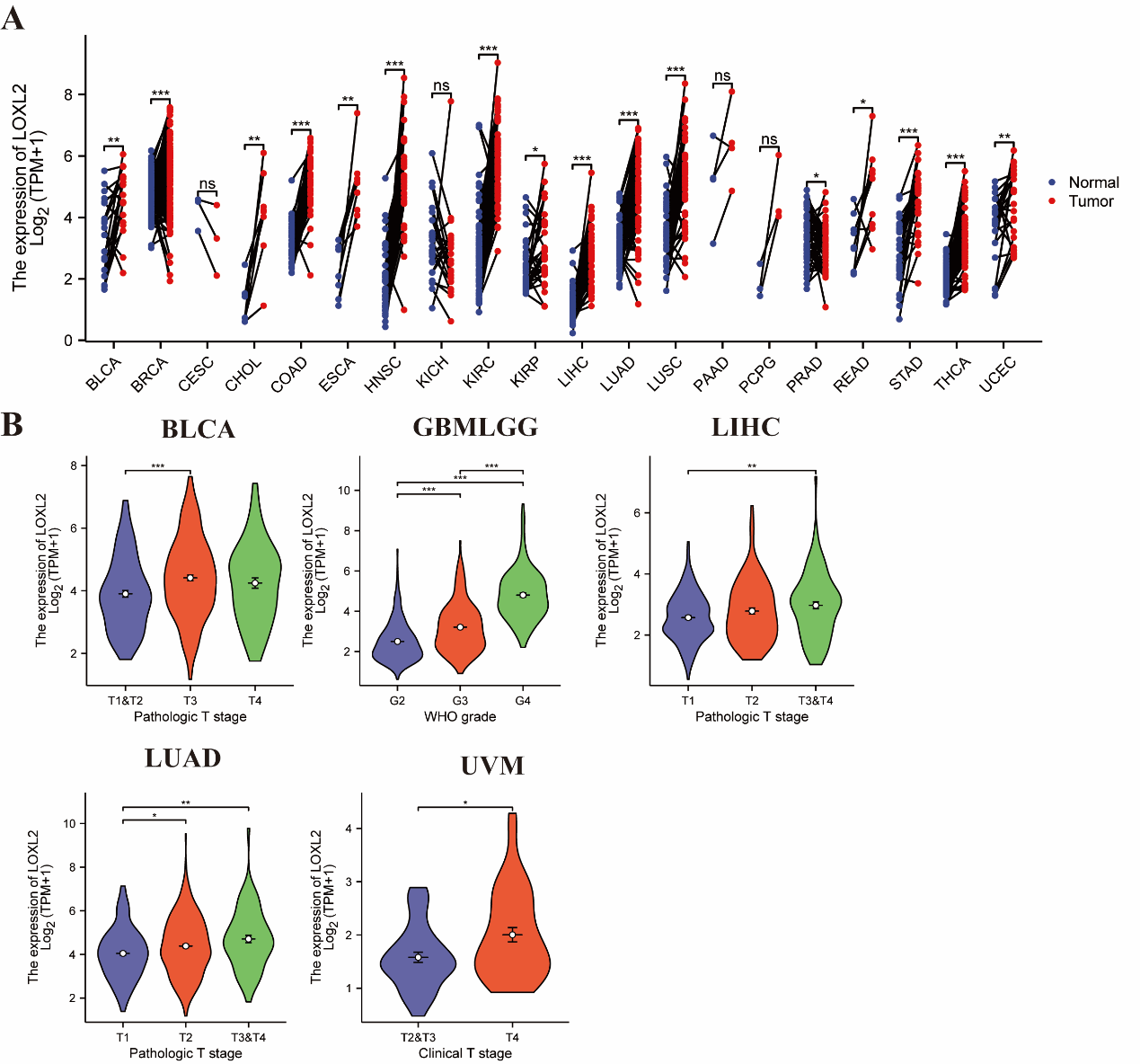


Figure S1 (A) For paired normal and tumor samples, mRNA expression levels of LOXL2 in different cancers were analyzed via the TCGA database; (B) Correlation of LOXL2 expression with clinicopathological stages of BLCA, GBMLGG, LIHC, LUAD, and UVM; *p < 0.05, **p < 0.01, and ***p < 0.001.


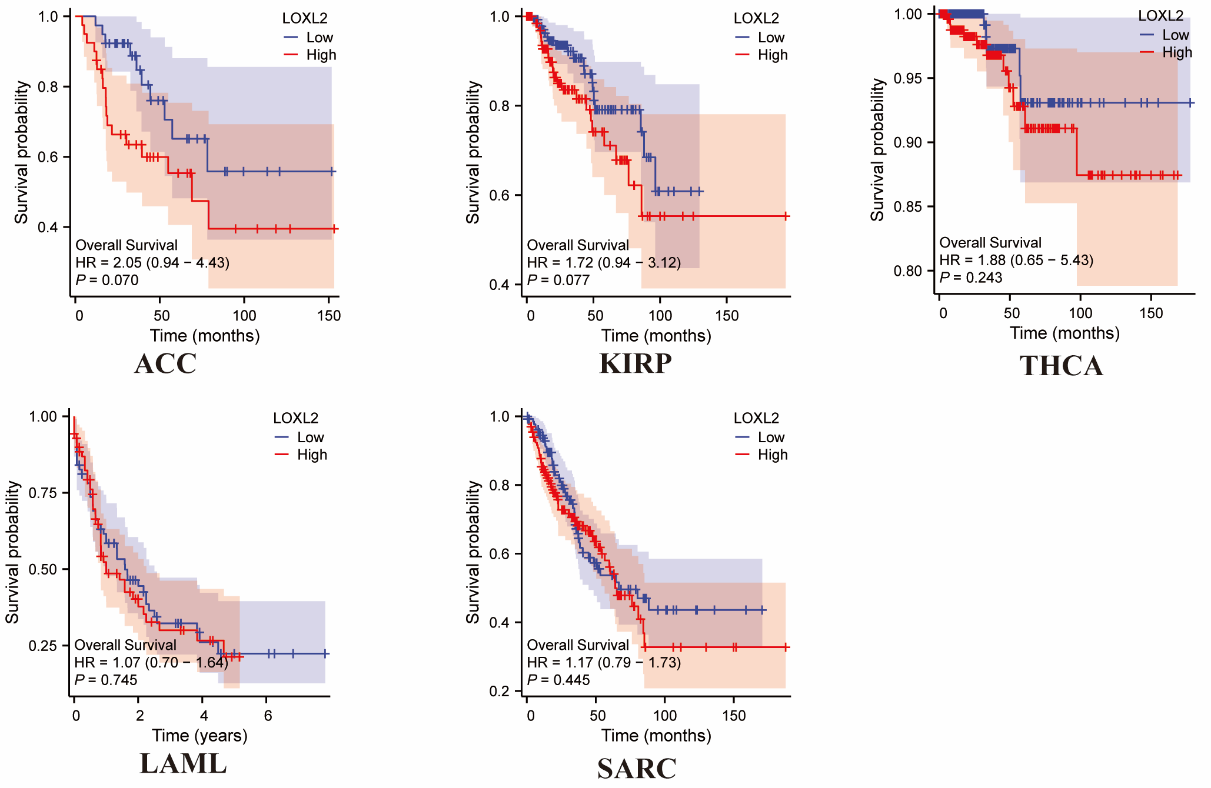


Figure S2 In the TCGA database, the Kaplan-Meier methodology was used to compare the expression of LOXL2 in different cancer types.


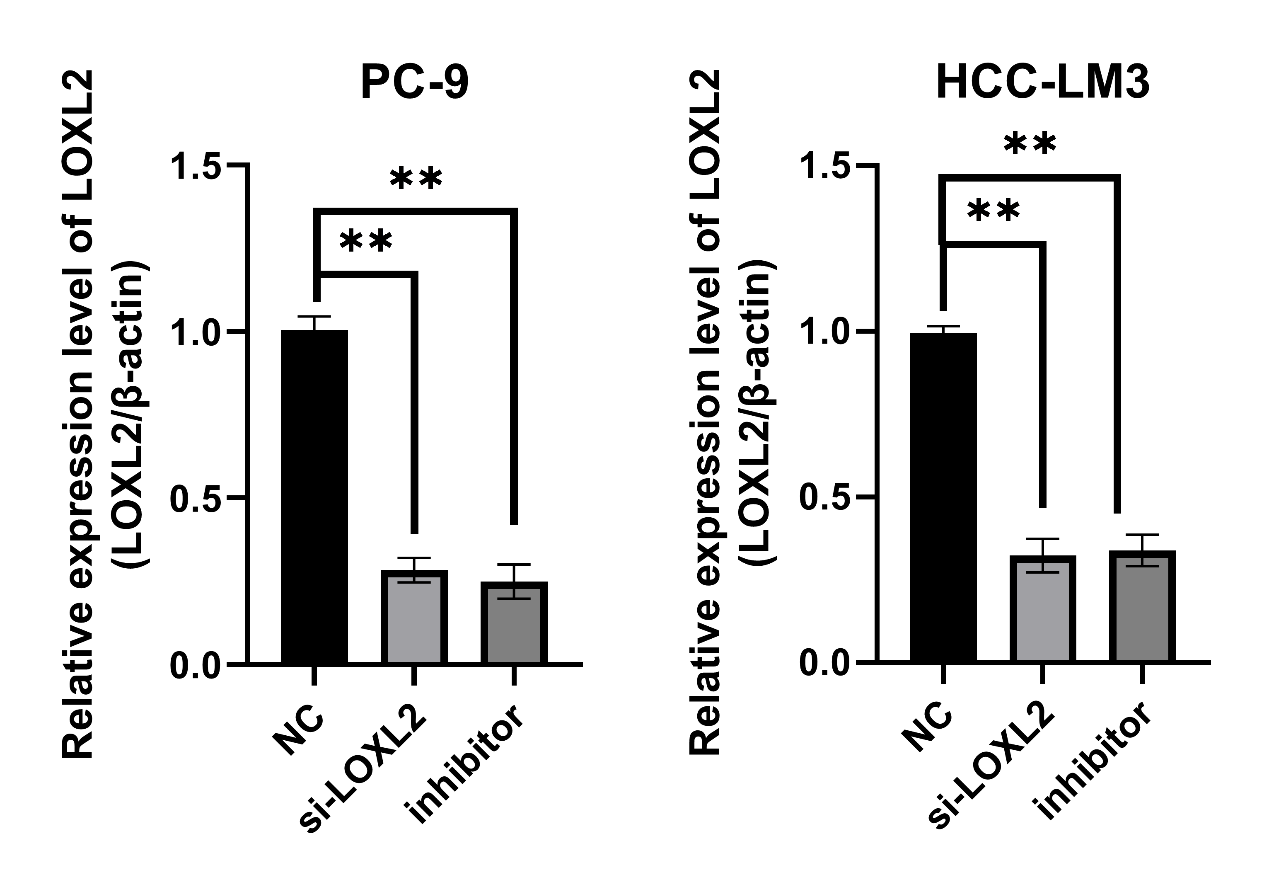


Figure S3 LOXL2 expression in PC-9 and HCC-LM3 cells treated with siRNA-LOXL2, inhibitor, and control. **p < 0.01.
